# Ionic Liquids as Interfacial Media for Metal-Free Electrochemical CO_2_ Reduction in Water

**DOI:** 10.1021/acssuschemeng.5c14224

**Published:** 2026-03-04

**Authors:** Welday Desta Weldu, Samuel Abidemi Oluwole, Solomon Owiredu, Nicole McGuire, Christian Agatemor

**Affiliations:** † Department of Chemistry, Waldorf University, Forest City, Iowa 50436, United States; ‡ Department of Chemistry, 5452 University of Miami, Coral Gables, Florida 33146, United States; § Department of Chemistry, 4517 Bucknell University, Lewisburg, Pennsylvania 18737, United States

**Keywords:** Electrochemical CO_2_ Reduction Reaction, Ionic Liquids, Carbon Dioxide, Metal-free CO_2_ Reduction Reaction, Aqueous CO_2_ Reduction Reaction

## Abstract

The electrochemical carbon dioxide reduction reaction (CO_2_RR) in water offers a sustainable pathway to mitigate carbon emissions while generating value-added chemicals. Most conventional CO_2_RR systems rely heavily on metal-based catalysts. Beyond traditional metal-based catalyst design, attention has increasingly shifted to understanding how the electrochemical microenvironment and the electrode–electrolyte interface influence CO_2_RR. Ionic liquids (ILs), widely regarded as green solvents, have previously been employed as electrolytes or cocatalysts in metal-catalyzed systems. Yet, the ability of ILs to facilitate CO_2_RR at metal-free interfaces in aqueous media remains underexplored. Here, we demonstrate that ILs polarize CO_2_ and facilitate CO_2_ electrochemical response at a glassy carbon interface under aqueous conditions, while simultaneously functioning as electrolytes. Spectroscopic, electrochemical, and computational analyses reveal that ILs interact with CO_2_, thereby increasing its dipole moment. This interaction suggests a favorable environment for CO_2_ polarization that correlates with the observed electrochemical response. The response efficiency depends on the chemical identity of the ILs, highlighting the tunability of this IL-based system. These findings redefine the functional role of ILs in CO_2_RR, establishing IL-induced molecular polarization as a potential strategy for promoting the reactivity of otherwise inert molecules. More broadly, this work introduces IL-driven dipole modulation as a general approach for enabling reactivity of nonpolar small molecules, with implications for sustainable chemical synthesis.

## Graphical abstract

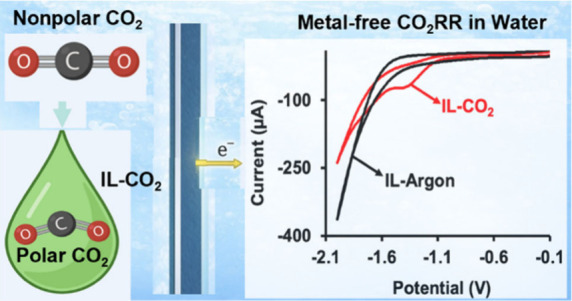

## Introduction

The electrochemical carbon dioxide reduction reaction (CO_2_RR) offers a sustainable route to mitigate carbon emissions while transforming CO_2_ into value-added chemicals.^1–17^ This transformation is attractive because it can proceed under ambient conditions and can be driven by renewable electricity, distinguishing it from energy-intensive thermochemical processes.^18^ As such, CO_2_RR has emerged as a key strategy for sustainable chemical manufacturing and carbon circularity. Despite significant progress, efficiently and scalably transforming CO_2_ into value-added chemicals via the electrochemical route remains a challenging task.^14,19^ CO_2_ is thermodynamically stable due to its linear geometry, strong C=O bonds, and zero dipole moment.^14^ As a result, its electrochemical transformation requires large overpotentials. Most existing CO_2_RR systems address this challenge using metal-based or molecular electrocatalysts that lower activation barriers and stabilize reaction intermediates.^20–25^ While effective, these catalysts often rely on expensive metals or complex synthesis routes, raising concerns about sustainability, cost, and scalability. Further, mass-transport limitations complicate aqueous CO_2_RR.^26–28^ The low solubility of CO_2_ in water restricts reactant availability at the electrode surface, suppressing reaction rates and favoring the competing hydrogen evolution reaction (HER).^26–28^ Considerable effort has therefore been devoted to catalyst design and electrode engineering to overcome these constraints. Nonetheless, beyond catalyst design, attention has increasingly shifted toward understanding how the electrochemical microenvironment and the electrode–electrolyte interface influence CO_2_ activation and reduction behavior.

Recent studies highlight the crucial role the electrochemical environment plays in CO_2_RR. For example, interfacial solvation significantly influences CO_2_ activation and reaction pathways.^29–32^ In particular, ionic liquids (ILs), a class of green solvents, have attracted attention for their ability to stabilize charged intermediates, enhance CO_2_ solubility, and modulate interfacial electric fields.^33–46^ Prior work has demonstrated that ILs dramatically improve CO_2_RR outcome when used as cocatalysts in conjunction with metal-based catalysts.^34,35^ However, existing studies largely treat ILs as auxiliary components, with limited attention to their direct influence on the electrochemical behavior of CO_2_. Their roles as electrolytes and as cocatalysts have been explored independently, with little emphasis on integrating these functionalities into a single, metal-free system. Moreover, while ILs are known to enhance CO_2_ solubility and intermediate stability,^36,37,41–43^ their ability to directly facilitate the CO_2_RR at metal-free interfaces in aqueous media remains underexplored.

Here, we address this gap by investigating ILs as interfacial media that facilitate CO_2_ electrochemical response on glassy carbon electrodes under aqueous conditions. We show that these ILs form organized domains in water that interact strongly with CO_2_, inducing molecular distortion and a substantial increase in CO_2_ dipole moment. This molecular perturbation suggests a favorable microenvironment that contributes to the observed CO_2_ electrochemical response. Rather than positioning ILs as alternatives to metal catalysts, this study examines CO_2_ electrochemical response under aqueous, IL-containing, metal-free conditions. By integrating electrolyte function with molecular polarization, this work provides insight into the role of IL-containing interface on glassy carbon. More broadly, these findings suggest that IL-driven dipole modulation is a promising strategy to facilitate the chemical reactivity of nonpolar molecules, opening new opportunities for sustainable electrochemical transformations.

## Materials and Methods

### General Information

Unless otherwise stated, all reagents and solvents were purchased from MilliporeSigma (Sigma-Aldrich) and used as received. These reagents and solvents include valeric acid (≥99%), choline bicarbonate (≈80 wt % in H_2_O), ferrocene (98%), tetrabutylammonium hexafluorophosphate (electrochemistry grade, ≥99.0%), and deuterium oxide (D_2_O, 99.9 atom % D). Geranic acid (technical grade, 85%) was obtained from MilliporeSigma and purified as described previously by recrystallization from acetone (HPLC grade, ≥99.8%) three times at −80 °C before use.^47^ Deionized (DI) water (18.2 MΩ·cm) obtained in-house and high-purity gases—carbon dioxide (CO_2_, ≥99.999%) and argon (Ar, ≥99.999%)—obtained from Airgas were used for electrochemistry experiments.

### Ionic Liquid (IL) Synthesis

The ILs were synthesized as described previously.^47^ Briefly, equimolar amounts of the carboxylic acid—valeric acid (for **IL 1**) or geranic acid (for **IL 2**)—and choline bicarbonate were mixed and stirred at room temperature until no more CO_2_ evolved (Figures 1a,b). The water was removed by rotary evaporation at 60 °C for 30 min, and the product was then dried and degassed (to remove residual CO_2_) in a vacuum oven at 60 °C for 48 h. The resulting ILs were characterized by ^1^H and ^13^C NMR and ATR-FT-IR spectroscopy as detailed below and shown in Figures S1–S4.

**Figure 1:**
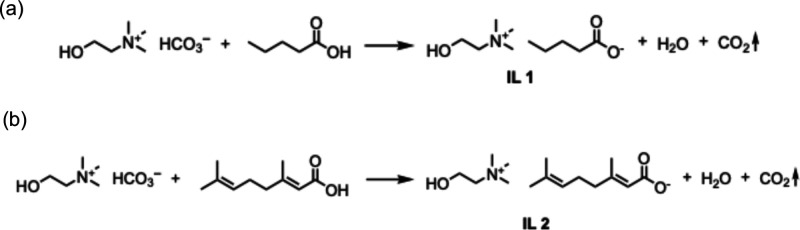
Synthesis of choline–carboxylate ionic liquids by acid–base neutralization of choline bicarbonate with (a) valeric acid (**IL 1**) or (b) geranic acid (**IL 2**). Equimolar amounts of the reactants were stirred at room temperature until cessation of CO_2_ evolution. Water was removed by rotary evaporation at 60 °C for 30 min, and the resulting ILs were dried under vacuum at 60 °C for 48 h.

### NMR and Infrared Spectroscopy

^1^H and ^13^C NMR spectra were acquired on a Bruker AV300 NMR spectrometer at ambient temperature. For structural elucidation, samples were prepared in DMSO-*d*_6_ (MilliporeSigma); chemical shifts are reported in parts per million (ppm) and referenced to the residual solvent signals (Figures S1–S4). To confirm structural integrity before and after CO_2_RR, samples were prepared in D_2_O (99.9 atom % D, MilliporeSigma). Attenuated total reflectance Fourier transform infrared (ATR-FT-IR) spectra were acquired using a PerkinElmer FT-IR spectrometer equipped with a diamond ATR accessory. The IL samples were analyzed directly by depositing a small aliquot onto the ATR crystal, ensuring full contact with the active surface before spectral acquisition. The Argon (Ar)- and CO_2_-sparged IL were used to probe IL–CO_2_ interactions; band positions for the carboxylate asymmetric stretch were analyzed for shifts indicative of interaction.

#### NMR Spectroscopic Data of **IL 1**

^1^H NMR (DMSO-*d*_6_, 300 MHz): δ 3.83 (CH_2_), 3.43 (CH_2_), 3.13 (C­(CH_3_)_3_), 1.85 (CH_2_), 1.38 (CH_2_), 1.22 (CH_2_), 0.81 (CH_3_) ppm. ^13^C NMR (DMSO-*d*_6_, 300 MHz): δ 176.55 (C = O), 67.30 (CH_2_), 54.99 (CH_2_), 53.10 (3x CH_3_), 38.31 (CH_2_), 28.77 (CH_2_), 22.47 (CH_2_), 14.01 (CH_3_) ppm.

#### NMR Spectroscopic Data of **IL 2**

^1^H NMR (DMSO-*d*_6_, 300 MHz): δ 5.52 (CH), 5.08 (CH), 3.86 (CH_2_), 3.45 (CH_2_), 3.14 (C­(CH_3_)_3_), 2.09–1.92 (CH_2_, CH_2_, CH_3_), 1.67 (CH_3_), 1.57 (CH_3_) ppm. ^13^C NMR (DMSO-*d*_6_, 300 MHz): δ 169.17 (C = O), 151.11 (C), 131.14 (C), 123.62 (CH), 120.57 (CH), 67.17 (CH_2_), 55.08 (CH_2_), 53.16 (3x CH_3_), 39.93 (CH_2_), 25.77 (CH_2_), 25.44 (CH_3_), 17.79 (CH_3_), 17.51 (CH_3_) ppm.

### Electrochemistry

Electrochemical measurements were performed on a C-3 Cell Stand (BASi Research Products) coupled to a PalmSens4 potentiostat controlled with PSTrace software. Unless otherwise noted, experiments employed a standard undivided three-electrode electrochemical cell equipped with a glassy carbon working electrode (3.0 mm diameter), a platinum counter electrode, and an Ag/AgCl reference electrode. All experiments were conducted using 40 mM solutions of **IL 1** and **IL 2**, a concentration selected based on preliminary screening that identified it as yielding the maximum CO_2_ reduction current. Before all experiments, aqueous solutions of the ILs were sparged with Ar or CO_2_ for 5 min to establish a defined gas environment. Cyclic voltammetry (CV) was recorded at 100 mV s^–1^ over 0 to −2 V vs Ag/AgCl to evaluate CO_2_RR. For mass-transport analysis, a rotating-disk electrode (BASi Research Products) was used for linear sweep voltammetry (LSV) at varied rotation rates, and Levich plots were constructed to confirm diffusion-controlled behavior. For ferrocene electrochemistry, a methanol/acetonitrile (1:1, v/v) solvent system, containing either 40 mM **IL 1** or 100 mM tetrabutylammonium hexafluorophosphate (TBAPF_6_) as the supporting electrolyte, and varying scan rates (80–10 mV s^–1^ over 0 to −2 V vs Ag/AgCl) was employed. Kinetic isotope-effect studies were conducted in D_2_O or 1:1 (v/v) D_2_O/H_2_O solutions of the IL sparged with Ar or CO_2_ gas. Chronoamperometric measurements were performed using the PalmSens4 potentiostat, with the applied potential for each IL set to the potential at which the maximum CO_2_ reduction current was observed. The current response was continuously monitored for 180 s under constant-potential conditions. Current densities were calculated by normalizing the measured cathodic current associated with CO_2_RR to the geometric surface area of the working electrode (3.0 mm diameter), allowing for a direct comparison of CO_2_ reduction activity across the two ILs.

### UV–Vis Spectroelectrochemistry

In situ spectroelectrochemical measurements were conducted under potentiostatic control, with spectra recorded as a function of time at a fixed applied potential. The spectroelectrochemical measurements were performed in a PalmSens4 photoelectrochemical cell, utilizing a quartz cuvette coupled to a PalmSens4 spectrophotometer. Optical and electrochemical data acquisition were controlled by AvaSoft and PSTrace software, respectively. The quartz cuvette was filled with an Ar- or CO_2_-sparged IL aqueous solution, into which a platinum mesh working electrode, a platinum counter electrode, and an Ag/AgCl reference electrode were inserted. A potential of −2 V vs Ag/AgCl, corresponding to the maximum cathodic current observed with a platinum mesh working electrode, was applied while UV–vis absorbance spectra were recorded in real time for 180 s to track the fate of the IL during CO_2_RR.

### Dynamic Light Scattering

Dynamic light scattering (DLS) measurements were performed using a Malvern Zetasizer Ultra (Malvern Panalytical, UK) operating in noninvasive backscatter (173°) geometry. Measurements were conducted at 22 °C, with samples equilibrated in the instrument for 60 s before data acquisition. Disposable polystyrene cuvettes (ZEN0040) were used, and automatic attenuation selection and measurement-position optimization were enabled to minimize multiple scattering and cell-to-cell variability. Each measurement consisted of three consecutive acquisitions, with five runs per acquisition (10 s per run), and the results were averaged. The dispersant was DI water, with refractive index and viscosity values set according to the instrument database (n = 1.33; η = 0.95 cP at 22 °C). The sample viscosity and refractive index were assumed to match those of the dispersant. Intensity autocorrelation functions were analyzed using the cumulants method to obtain the Z-average hydrodynamic diameter and polydispersity index (PdI). In addition, intensity-weighted size distributions were calculated using the instrument’s regularized non-negative least-squares inversion algorithm (Zetasizer Xplorer, normal resolution model). The Zetasizer platform can detect scattering features over an approximate size range of ∼0.3 nm to ∼10 μm, depending on the sample’s optical properties and scattering contrast.^48^ Nevertheless, the reported size distributions were interpreted as effective hydrodynamic scattering domains rather than discrete particle dimensions. In systems containing amphiphilic ILs, such distributions reflect changes in mesoscale organization or transient scattering heterogeneities rather than well-defined particulate species.^49–51^ For each IL, aqueous solutions (40 mM) were prepared using DI water. To minimize scattering artifacts arising from transient gas microbubbles, solutions were handled in capped, low-headspace tubes, briefly centrifuged (500 g, 1 min), and equilibrated at room temperature for 20 min before analysis. During cuvette loading, pipet tips were prewetted, and samples were dispensed slowly along the cuvette wall to minimize air entrainment. This was followed by an additional 2 min of equilibration before data acquisition. These precautions ensured that observed changes in scattering profiles reflect intrinsic differences in solution structure rather than artifacts arising from bubble formation or handling. Measurements were performed under three parallel conditions using identical instrumental settings: (i) IL aqueous solution, (ii) Ar-sparged IL aqueous solutions, and (iii) CO_2_-sparged IL aqueous solutions.

### Density Functional Theory (DFT) Calculations

The DFT calculations were carried out with Gaussian 16 ^52^ on the University of Miami Pegasus cluster. Gas-phase cluster models made up of CO_2_ and the ILs were built in GaussView 6.1.1. Multiple starting geometries were generated by preorienting hydrogen-bond donor–acceptor pairs (D–H···A) within the ion pair with CO_2_ to favor interaction (D–H···A ≈ 2.5 Å, ∠D–H···A ≈ 180°). The CO_2_ was positioned in representative alignment motifs: end-on to a carboxylate oxygen, side-on above the carboxylate region, proximal to the cholinium hydroxyl group, and adjacent to the quaternary ammonium group. For each motif, CO_2_ was initially positioned ∼2.8–3.8 Å from the nearest site, and both parallel and perpendicular orientations of the CO_2_ molecular axis relative to local charge vectors were sampled before full, unconstrained optimization. Geometry optimizations and harmonic frequency analyses were performed at B3LYP/6-311G level, and only true local minima (no imaginary frequencies) were retained. As a reference, isolated gas-phase CO_2_ (no IL present) was also optimized and characterized at B3LYP/6-311G level to provide a baseline dipole moment and O–C–O angle for comparison. For each system, the lowest-energy minimum was used to extract the CO_2_ dipole moment and O–C–O bond angle, which were then compared with those of free gas-phase CO_2_ optimized at the same level.

## Results and Discussion

We first established whether the ILs provide adequate electrolyte functionality. Cyclic voltammetry (CV) and linear sweep voltammetry (LSV) were therefore performed using ferrocene as a benchmark outer-sphere redox probe. A methanol/acetonitrile (1:1, v/v) solvent system was selected for this experiment to ensure adequate solubility of both the IL and ferrocene. Ferrocene is suited for this purpose because its electrochemistry is well-established and sensitive to ionic conductivity, mass transport, and coupled chemical reactions.^53^ When **IL 1** was used as the sole supporting electrolyte with a glassy carbon working electrode, without added TBAPF_6_, ferrocene exhibited a well-defined and reversible one-electron redox couple (Figure 2a). The peak positions and symmetry were consistent with diffusion-controlled electron transfer and minimal ohmic distortion.^53,54^ This behavior demonstrates that **IL 1** provides sufficient ionic mobility and charge compensation to support homogeneous electrochemical reactions. Rotating disk electrode LSV measurements further confirmed this conclusion. The limiting current increased systematically with increasing rotation rate (Figures 2b and 2c), consistent with Levich behavior, implying the absence of sluggish kinetics, adsorption, or follow-up chemical reactions over the investigated potential range.^55^ These results indicate that mass transport and charge transfer are not hindered during this electrochemical measurement. Importantly, these electrochemical benchmarking experiments demonstrate that **IL 1** functions reliably as an electrolyte.

**Figure 2:**
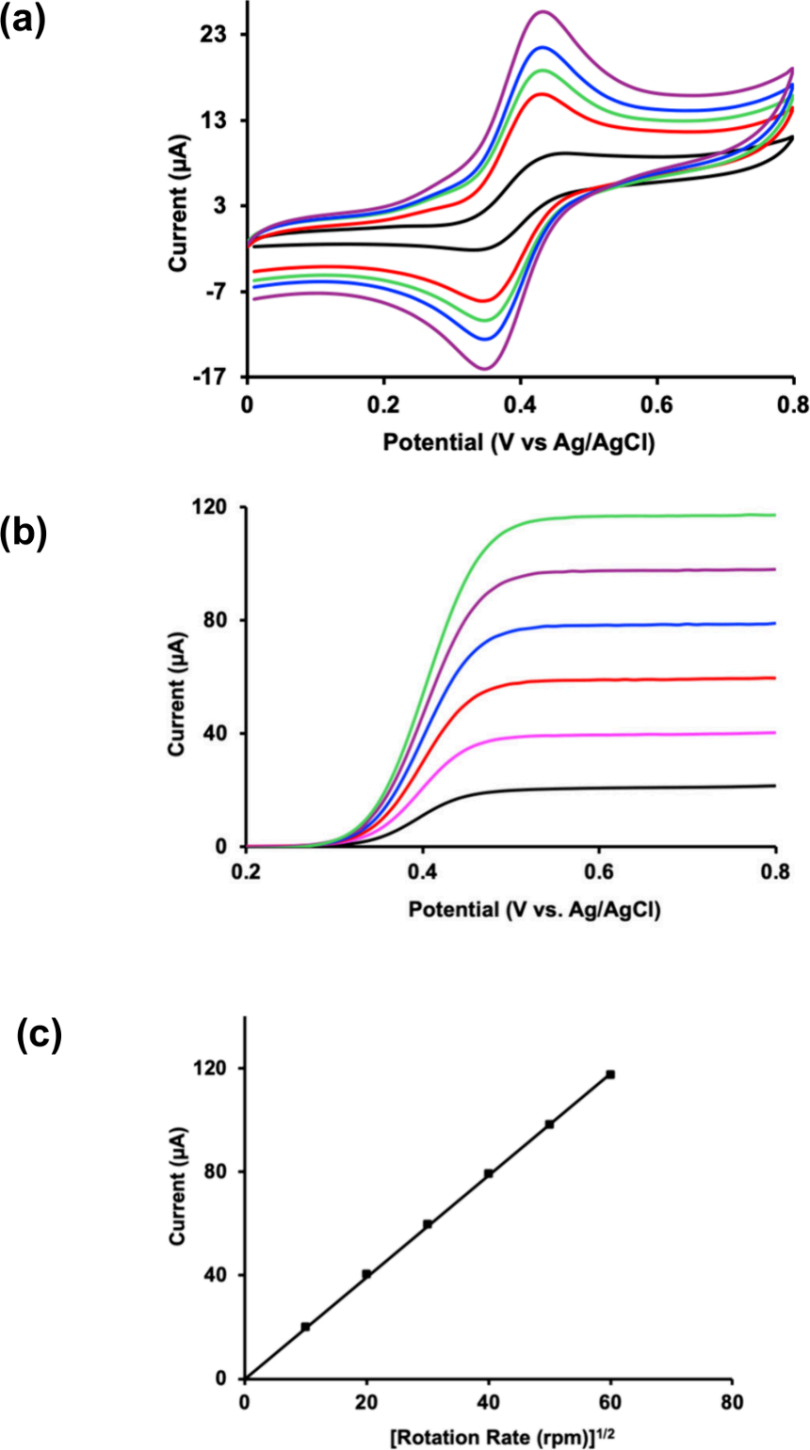
Electrochemical behavior of ferrocene in **IL 1** serving as the supporting electrolyte. (a) Cyclic voltammograms of ferrocene recorded at varying scan rates (purple: 0.08 V s^–1^; blue: 0.06 V s^–1^; green: 0.05 V s^–1^; red: 0.04 V s^–1^; black: 0.01 V s^–1^), showing scan-rate-dependent redox response. (b) Rotating disk electrode linear sweep voltammograms obtained at different rotation rates (green: 3600 rpm; purple: 2500 rpm; blue: 1600 rpm; red: 900 rpm; cyan: 400 rpm; black: 100 rpm). (c) Levich plot showing the linear relationship between limiting current and the square root of the electrode rotation rate. Electrochemical measurements were performed using a glassy carbon working electrode, a platinum wire counter electrode, and an Ag/AgCl reference electrode in a 40 mM methanol/acetonitrile (1:1, v/v) solution of **IL1** as the supporting electrolyte.

ILs, particularly those containing imidazolium cations, are widely reported as cocatalysts in metal-catalyzed CO_2_RR.^34,35,43^ In these systems, ILs enhance CO_2_RR by stabilizing charged intermediates, modifying the electric double layer, or increasing the local CO_2_ concentration near the electrode surface.^34,35,43^ However, the extent to which ILs influence the CO_2_RR on metal-free interfaces remains comparatively underexplored. Previous studies have shown that ILs can interact with CO_2_ through electrostatic interactions, hydrogen bonding, and local electric-field effects.^34,35,56–58^ These interactions can partially polarize CO_2_ and stabilize its reduced forms, the CO_2_ radical anion (CO_2_^•–^). Indeed, Rosen et al. demonstrated that imidazolium-based ILs promote CO_2_RR by stabilizing CO_2_^•–^ at electrode interfaces.^34,35^ Also, Sun et al.^33^ and Ju et al.^38^ further showed the importance of IL-induced interfacial structuring and solvation effects in lowering the energetic barrier for CO_2_RR. Together, these studies demonstrate that ILs stabilize reduced CO_2_ intermediates and, importantly, motivate the hypothesis that ILs polarize CO_2_, facilitating CO_2_RR on a metal-free interface.

Guided by this hypothesis, CV was employed to investigate whether **IL 1** promotes a CO_2_ electrochemical response under metal-free conditions while simultaneously serving as the supporting electrolyte. Experiments were conducted in an undivided three-electrode electrochemical cell equipped with a glassy carbon working electrode, a platinum counter electrode, and an Ag/AgCl reference electrode. Measurements were performed using 40 mM aqueous solutions of **IL 1**, identified as the optimal concentration from preliminary screening, under Ar- and CO_2_-sparged conditions and without added metal catalysts or supporting electrolytes. Under the CO_2_-sparged condition, the cyclic voltammogram shows a distinct irreversible cathodic peak at −1.3 V vs Ag/AgCl (Figure 3a), consistent with CO_2_RR.^59–61^ This feature was absent in the Ar-sparged control, which instead showed a cathodic peak associated with HER,^8^ as expected at negative potentials in aqueous systems. The appearance of this cathodic peak only under the CO_2_ condition indicates that **IL 1** alters the electrochemical environment in a manner that promotes CO_2_RR. Crucially, this reduction feature was absent when **IL 1** was replaced by either of its molecular precursors, choline or valeric acid (Figure S5). These findings indicate that the reduction behavior is not an intrinsic property of the individual precursor. Instead, the results highlight the importance of the IL’s ionic environment. Collectively, these results demonstrate that the IL establishes a unique interfacial environment, through ion pairing, self-organization, and local ionic structuring, that promotes the observed metal-free CO_2_ electrochemical response.

**Figure 3:**
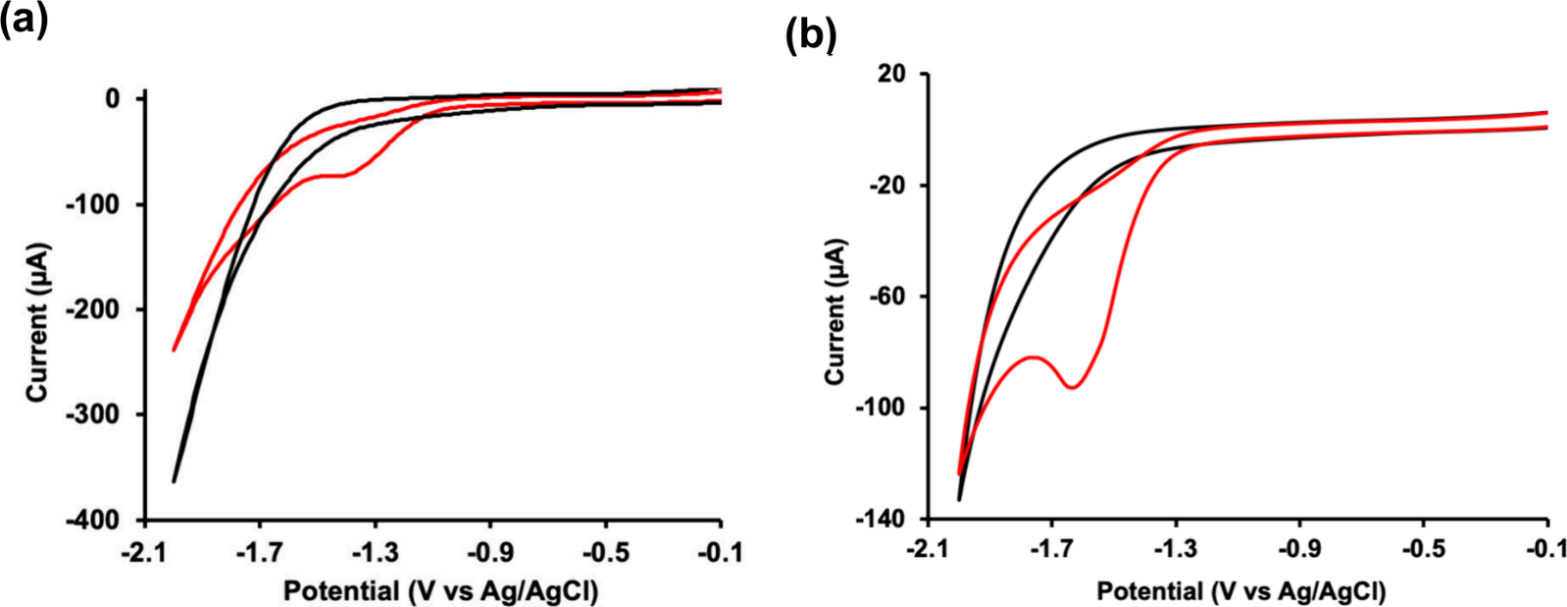
Cyclic voltammograms illustrating CO_2_RR in aqueous IL electrolytes. (a) 40 mM **IL 1** and (b) 40 mM **IL 2** under Ar-sparged (black) and CO2-sparged (red) conditions. The appearance of an additional cathodic feature under CO_2_ relative to Ar indicates CO_2_-dependent electrochemical behavior. Electrochemical measurements were performed using a glassy carbon working electrode, a platinum wire counter electrode, and an Ag/AgCl reference electrode in 40 mM aqueous solutions of the IL, without added supporting electrolyte or metal catalyst.

Based on established CO_2_RR studies, the irreversible reduction peak at −1.3 V vs Ag/AgCl (Figure 3a) is attributed to the one-electron reduction of CO_2_ to CO_2_^*–^.^59–61^ Formation of this species represents the initial and energetically demanding step in CO_2_RR and is widely recognized as a key bottleneck in both metal-catalyzed and metal-free systems. Once generated, CO_2_^•–^ undergoes subsequent chemical or electrochemical transformations, depending on the electrode material, solvent, and reaction conditions. Notably, the potential required for CO_2_^•–^ formation in this system was substantially less negative than values reported for conventional electrolyte systems. For example, CO_2_ reduction in DMSO using tetraethylammonium perchlorate occurs at approximately −1.60 V vs Ag/AgCl on platinum electrodes and −1.88 V vs Ag/AgCl on glassy carbon electrodes.^60^ The lower potential observed here suggests that **IL 1** more effectively facilitates CO_2_RR, potentially by stabilizing the incipient CO_2_^•–^ through electrostatic interactions and local solvation effects. It is important to emphasize that **IL 1** is not proposed to function as a molecular electrocatalyst. Instead, the results indicate that **IL 1** facilitates the observed CO_2_ electrochemical response by establishing a favorable ionic and interfacial environment in water, even in the absence of metal catalysts. This distinction aligns with current understanding of IL-assisted CO_2_RR and avoids overinterpretation in the absence of product analysis and turnover metrics.

The properties of ILs arise from their constituent ions,^62^ making IL composition a critical lever for tuning CO_2_RR performance. Prior work shows that changing the IL cation or anion reshapes the electrical double layer, alters interfacial solvation, and stabilizes early reduced CO_2_ intermediates, thereby shifting CO_2_RR onset potentials and modulating competition with HER.^56,58^ In particular, systematic screening studies in IL-containing electrolytes report that anion identity and ion-pairing strength influence activity and selectivity,^58^ while cation structure governs interfacial organization and the effective stabilization of CO_2_-derived intermediates.^56^ Guided by this framework, we replaced valerate in **IL 1** with another anion, geranate, to form **IL 2** (Figure 1). This substitution probes how IL composition modulates the electrochemical response, and also provides an additional control to confirm that the observed behavior arises from the intrinsic IL environment rather than from the individual molecular precursors. Under CO_2_-sparged conditions, **IL 2** exhibited a cathodic reduction peak at −1.6 V vs Ag/AgCl, a shift to a more negative potential relative to **IL 1** (Figure 3b). Further, **IL 2** reached a larger cathodic current density (−2.24 mA cm^–2^) than **IL 1** (−0.53 mA cm^–2^), but only at a substantially more negative potential, indicating that **IL 2** requires a greater driving force to facilitate the CO_2_ electrochemical response. Under Ar-sparged conditions, **IL 2** showed no CO_2_-related reduction peak and displayed a lower HER current than **IL 1** (Figure 3), consistent with stronger HER suppression. Taken together, these results reveal a clear composition-dependent trade-off: although **IL 2** supports higher absolute cathodic currents and suppresses HER, **IL 1** promotes CO_2_ electrochemical response at significantly lower overpotentials. This distinction underscores the critical role of ionic composition in governing electrochemical responses and highlights how subtle variations in ion pairing and local solvation profoundly influence CO_2_ reduction behavior.

The ability of **IL 1** and **IL 2** to facilitate CO_2_RR raises important questions about the underlying mechanism. In IL-mediated chemical transformations, including CO_2_RR, where ILs function as cocatalysts, the consensus is that ILs interact with substrates through noncovalent intermolecular interactions, such as electrostatic forces, hydrogen bonding, or local solvation effects, to lower activation barriers.^16,20,43^ In aqueous environments, additional complexity arises from the propensity of amphiphilic ILs, such as **IL 1** and **IL 2**, to undergo self-association and mesoscale organization rather than remaining as discrete ion pairs. Indeed, accumulating evidence demonstrates that amphiphilic ILs in water form a range of self-assembled micro- and nanostructures, including micelle-like aggregates, vesicles, and other dispersed ionic domains.^63–74^ Importantly, several studies have shown that exposure to CO_2_ induces pronounced and reversible changes in these self-assembled structures, as detected by dynamic light scattering (DLS), small-angle scattering, spectroscopic, and microscopic techniques.^63,71^ These CO_2_-induced reorganizations are attributed to changes in IL–CO_2_ interactions, arising from altered ion pairing, hydrogen-bonding networks, and local polarity, rather than from irreversible chemical transformations.^63,71^ Collectively, these findings establish that CO_2_ acts as a stimulus that modulates IL microstructure in aqueous systems. Based on this context, we hypothesized that IL–CO_2_ interaction reorganizes IL self-assembled microstructure in water and promotes CO_2_ polarization. To test this hypothesis, we employed DLS to assess changes in the effective hydrodynamic scattering domains of the ILs in water upon exposure to CO_2_. This approach provides insight into CO_2_-induced reorganization of the IL microstructure and qualitatively corroborates previously reported CO_2_–IL interactions.^63,71^ The DLS measurements reveal distinct changes in the mesoscale organization of the ILs under different gas conditions. In their neat form, both **IL 1** and **IL 2** exhibited broad scattering distributions (Figures 4a and 4b), consistent with previous reports,^63,71^ that amphiphilic ILs form dynamic, loosely associated domains in aqueous media. These features reflect collective density fluctuations rather than discrete, well-defined particles. Upon sparging with Ar, the scattering profiles remain qualitatively similar but exhibit a modest redistribution in intensity, indicating that gas bubbling alone perturbs the system only slightly. In contrast, exposure to CO_2_ produces pronounced, reproducible changes in the scattering behavior (Figures 4a and 4b). For **IL 2**, CO_2_ induces a shift in the dominant scattering contribution toward larger effective scattering length scales, whereas **IL 1** exhibits a redistribution toward smaller effective scattering domains. These divergent trends indicate that CO_2_ interacts differently with each IL, altering their mesoscale organization in distinct ways. Because the DLS data report intensity-weighted scattering rather than discrete particle dimensions,^51^ these changes are best interpreted as CO_2_-induced reorganization of the IL’s environments rather than formation or dissolution of specific particulate species. The observed behavior is consistent with modulation of ion–ion interactions, hydrogen bonding, and local polarity upon CO_2_ uptake as previously reported.^63,71^ Collectively, these results indicate that CO_2_ alters the mesoscale organization of the ILs in a composition-dependent manner, providing qualitative evidence of CO_2_–IL interactions, which can influence the physicochemical environment relevant to electrochemical processes. To further probe the presence of CO_2_–IL interactions experimentally, we employed attenuated total reflectance infrared (ATR-IR) spectroscopy. The ATR-IR spectrum of CO_2_-sparged **IL 2** revealed a slight but reproducible blue shift in the asymmetric stretching vibration of the carboxylate group. Specifically, the band centered at approximately 1552 cm^–1^ in Ar-sparged **IL 2** shifted to 1556 cm^–1^ upon CO_2_ exposure (Figure 4c, inset). A comparable blue shift was observed for **IL 1** under CO_2_-sparged conditions (Figure S6). Such shifts to higher wavenumber are commonly associated with modest bond stiffening and are consistent with interaction-induced electronic redistribution within the carboxylate group.^73^

**Figure 4:**
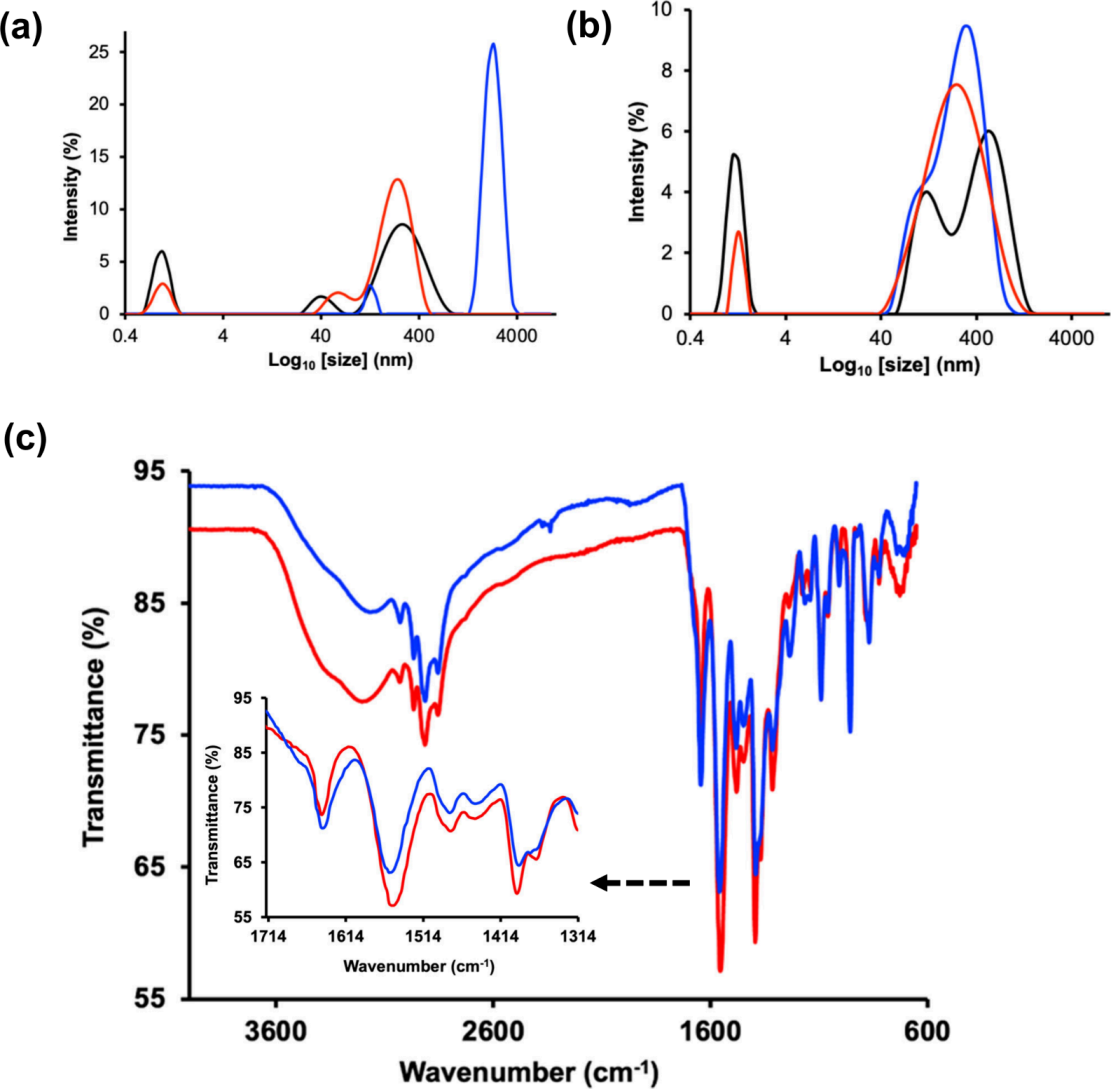
Evidence for CO_2_–ionic liquid interactions probed by DLS and infrared spectroscopy. (a,b) Intensity-weighted DLS distributions of 40 mM **IL 2** (a) and **IL 1** (b) under different conditions: neat IL (black), Ar-sparged (red), and CO_2_-sparged (blue). Exposure to CO_2_ shifts the scattering distributions relative to the neat and Ar-treated samples, indicating CO_2_-induced reorganization of the IL in water. (c) ATR–FTIR spectra of **IL 2** recorded under Ar (red) and CO_2_ (blue) conditions. CO_2_ exposure alters the carboxylate stretching region, consistent with changes in the local chemical environment. Insets highlight the evolution of the ν_(COO^–^)_ region upon gas exposure.

Interactions between CO_2_ and its local environment are known to modulate CO_2_ reactivity by perturbing its electronic structure and lowering activation barriers. Prior studies have demonstrated that intermolecular interactions, including electrostatic forces, hydrogen bonding, and van der Waals interactions, alter the polarity, bond strength, and molecular geometry of CO_2_, facilitating its chemical transformations.^75–79^ Building on this, we hypothesized that CO_2_–IL interactions polarize CO_2_ and tested this hypothesis using density functional theory (DFT) calculations with the B3LYP functional and a 6-311G basis set. These calculations were used to examine changes in the CO_2_ dipole moment, an indicator of molecular polarization that provides insight into the likelihood of molecular activation. In the absence of IL, CO_2_ exhibited a dipole moment of 0 D, consistent with its linear and symmetric geometry (Table 1). In contrast, in the presence of **IL 1** or **IL 2**, CO_2_ developed a nonzero dipole moment ranging from 0.29 to 1.04 D (Table 1), indicating polarization. The optimized structures suggest that these interactions are noncovalent, likely dominated by van der Waals and electrostatic interactions, as reflected by the relatively long intermolecular distances between CO_2_ and the IL components (Figures 5a and 5b).^80^ Importantly, the calculations also reveal that CO_2_ undergoes a distortion from linearity upon interaction with the ILs. Whereas free CO_2_ retains a bond angle of 180°, interaction with **IL 2** results in a pronounced bending of the molecule to approximately 134° (Table 1). Similar distortions were observed for **IL 1**. Such geometric distortion breaks the molecular symmetry of CO_2_, providing a plausible origin for the induced dipole moment. A bent, polarized CO_2_ is expected to exhibit a lower activation barrier for electron transfer. Collectively, these findings demonstrate that the ILs interact with and polarize CO_2_, providing a plausible mechanistic basis for the observed IL-mediated CO_2_ electrochemical response.

**Table 1:** Calculated Dipole Moments and O–C–O Bond Angles for Free CO_2_ and CO_2_ Interacting with **IL 1** and **2**, as Obtained from DFT Calculations. Interaction with the IL Polarized CO_2_ and Bent the Molecule from Linear Geometry

Species	Dipole moment (μ, D)	Bond angle (deg)
CO_2_	0	180
**IL 1**-CO_2_	0.30	168
**IL 2**-CO_2_	1.04	134

**Figure 5:**
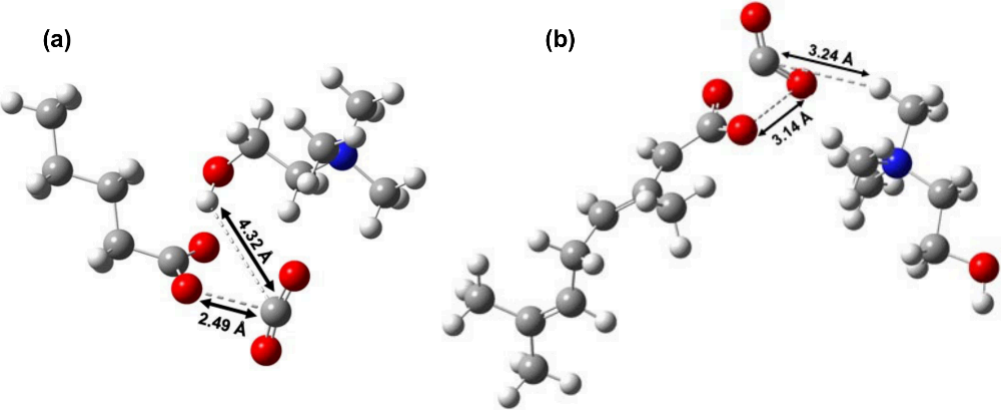
Optimized geometries of CO_2_ interacting with (a) **IL 1** and (b) **IL 2** obtained from DFT calculations. The models illustrate the distinct binding motifs and intermolecular interactions between CO_2_ and each IL, including changes in CO_2_ bond geometry and proximity to functional groups of the IL. Distances (Å) highlight key intermolecular distances.

Proton-coupled electron transfer (PCET) is a central feature of CO_2_RR in aqueous media, in which electron transfer to CO_2_ is intrinsically coupled to proton transfer in either a concerted or stepwise manner.^32,81–84^ To assess whether proton transfer is integral to the IL-mediated CO_2_ electrochemical response on glassy carbon interface observed here, we examined the electrochemical behavior in deuterium oxide (D_2_O) as a direct probe of kinetic isotope effects. Replacing H_2_O with D_2_O is expected to perturb PCET-limited processes due to the stronger D–O bond and the associated decrease in proton transfer rates. Consistent with this expectation, the CO_2_-related reduction feature observed for **IL 2** in H_2_O was no longer detectable under CO_2_-sparged conditions in D_2_O (Figure 6a). This pronounced isotope effect suggests that proton transfer is coupled to the electron-transfer step underlying the observed disappearance in D_2_O. Introducing H_2_O into D_2_O (1:1 v/v) partially restores the reduction peak, but with a diminished cathodic current (Figure 6a), reinforcing the role of proton transfer in the reduction process. The CO_2_ electrochemical response in **IL 1** showed a similar trend when measured in D_2_O. The reduction peak shifted to a more negative potential, and the cathodic current decreased relative to when measured in H_2_O (Figure 6b), consistent with a kinetically hindered proton-transfer step. The systematic suppression and recovery of the reduction feature upon isotopic dilution strongly suggest that proton transfer is a key contributor to the electrochemical response. Together, these isotope-dependent effects show that proton transfer plays a role in the IL-mediated CO_2_ electrochemical response observed on the glassy carbon under aqueous conditions. Although these results do not define the precise sequence or concerted nature of the PCET steps, they demonstrate that proton availability and proton-transfer kinetics directly govern these ILs’ ability to facilitate CO_2_ electrochemical response at a glassy carbon interface.

**Figure 6:**
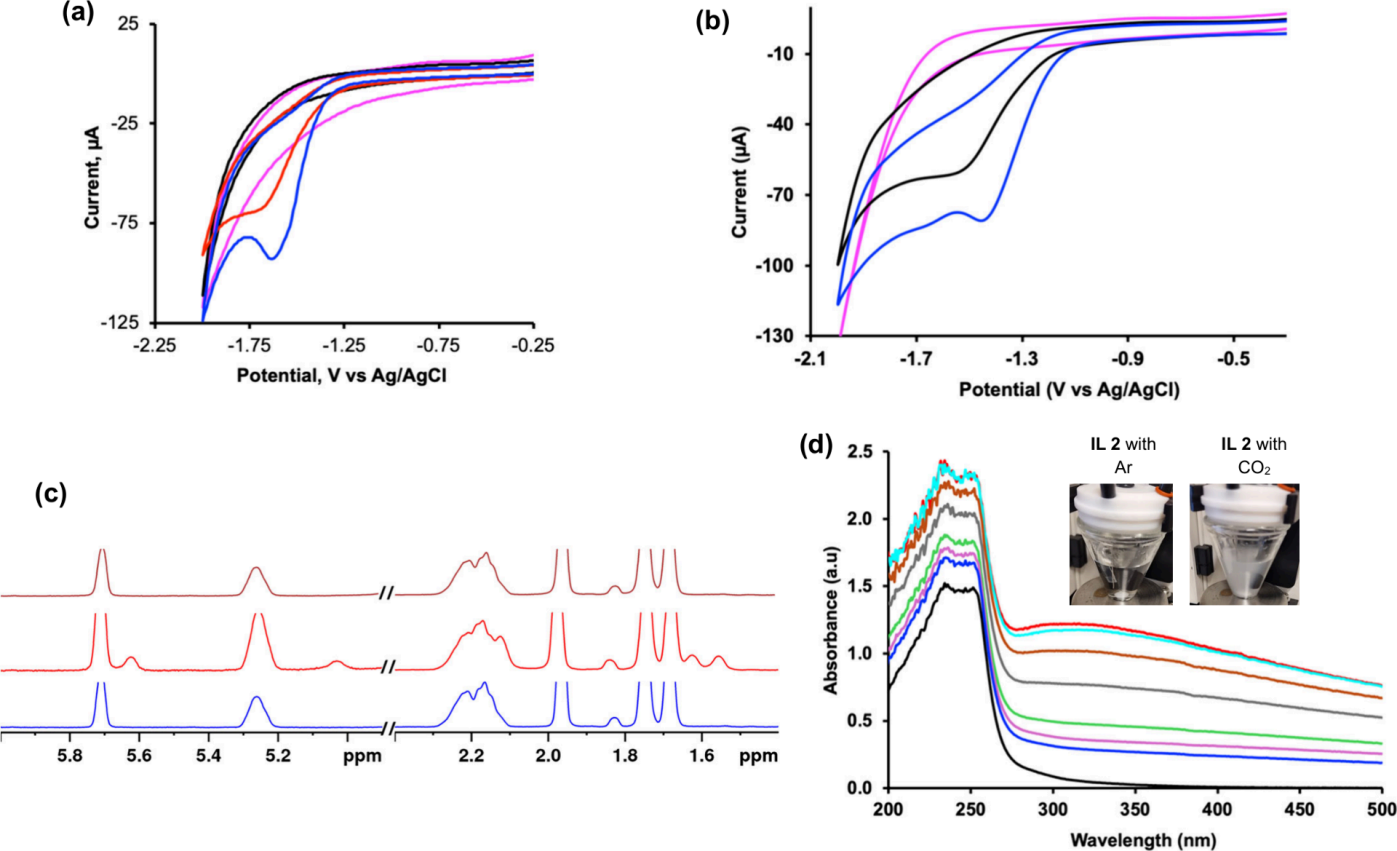
Electrochemical and spectroscopic characterization reveal kinetic isotope effect and inertness of IL during CO_2_RR. (a) Cyclic voltammograms of **IL 2** recorded in D_2_O under different gas environments: CO_2_-sparged (black), Ar-sparged (magenta), D_2_O/H_2_O (1:1, v/v) under CO_2_ (red), and H_2_O under CO_2_ (blue). (b) Cyclic voltammograms of **IL 1** recorded in D_2_O under CO_2_ (black), D_2_O under Ar (magenta), and H_2_O under CO_2_ (blue). Measurements were performed using a glassy carbon working electrode, a platinum wire counter electrode, and an Ag/AgCl reference electrode at a scan rate of 100 mV s^–1^. (c) ^1^H NMR spectra of **IL 2** showing spectra collected under Ar (blue), under CO_2_ prior to CO_2_RR (red), and after CO_2_RR followed by Ar purging (brown), demonstrating the inertness of the IL structure. (d) In situ UV–vis spectra recorded during CO_2_RR showing time-dependent spectral changes (black: *t* = 0 ms under Ar; red: CO_2_-sparged at *t* = 0 ms; cyan: 60 ms; brown: 80 ms; gray: 100 ms; green: 120 ms; purple: 140 ms; blue: 160 ms), consistent with reversible electronic perturbations associated with CO_2_ interaction. (d insert) Pictures of **IL 2** aqueous solution sparged with Ar or CO_2_. The **IL 2** solution turned turbid upon exposure to CO_2_.

A critical question is whether the ILs undergo irreversible redox chemistry and act as sacrificial reagents during CO_2_RR conditions. We addressed this question using complementary ^1^H NMR spectroscopy, in situ UV–vis spectroelectrochemistry, and chronoamperometry to track the chemical and electrochemical stability of the ILs during CO_2_ electrochemical response. ^1^H NMR spectrum of **IL 2** recorded after CO_2_ sparging revealed additional resonances that were absent under argon (Figure 6c). The appearance of a new resonance in the ^1^H NMR spectrum upon CO_2_ exposure indicates the formation of a distinct chemical environment for a subset of IL protons, suggesting interaction between the IL and CO_2_ rather than the formation of a new covalent species. These resonances disappeared upon argon purging of the post-CO_2_RR solution. Importantly, the spectra collected before and after CO_2_RR were identical (Figure 6c), demonstrating that the IL retained its molecular structure throughout the CO_2_RR process. These observations indicate that the new NMR peaks arise from reversible CO_2_–IL interactions, rather than from a permanent chemical modification or degradation.

We complemented the NMR experiment with in situ UV–vis spectro-electrochemistry using a platinum mesh working electrode to monitor changes in the optical response of **IL 2** under CO_2_RR conditions. Notably, CO_2_-associated cathodic currents recorded with the platinum mesh working electrode in the spectroelectrochemical setup occurred at more negative potentials (∼−2.0 V vs Ag/AgCl) than those observed with the glassy carbon electrode (Figures 3a and 3b), indicating that the IL-mediated CO_2_ electrochemical response is electrode-dependent. Further, under argon, **IL 2** exhibited absorption bands near ∼236 and ∼252 nm, consistent with a previously reported carbonyl-centered electronic transitions.^85^ Upon CO_2_ sparging, before applying an electrochemical potential (*t* = 0), the spectrum changed, showing increased UV absorbance and the emergence of a broad feature extending toward ∼ 315 nm (Figure 6d). In contrast, this feature was absent in the UV–vis spectra of the Ar-sparged IL (Figure S7). This difference between Ar- and CO_2_-sparged IL establishes CO_2_ exposure as the origin of the new optical response. During CO_2_RR, the intensity of this broad feature decreased as the reaction progressed, with the spectrum gradually returning to the profile observed under Ar. The reversibility and CO_2_ dependence of the UV–vis response argue against irreversible IL consumption during CO_2_RR. We interpret these spectral changes conservatively. We do not assign the emergent absorption to a discrete catalytic intermediate or a specific absorbing species. Instead, we posit that the data indicate reversible CO_2_-induced perturbations of the IL’s electronic and mesoscale environment. For example, CO_2_–IL interactions can redistribute electron density within the carboxylate framework, consistent with the interaction-induced blue shifts observed in ATR-IR spectra (Figure 4c and Figure S6) and the polarization predicted by DFT calculations (Table 1 and Figure 5). In addition, CO_2_ sparging visibly increased solution turbidity (Figure 6d insert), indicating mesoscale reorganization that can enhance apparent absorbance through light scattering, particularly at longer wavelengths. Such scattering-assisted optical changes are well documented in self-organized ionic and amphiphilic systems and do not require the formation of new chromophores.^86,87^ Consistent with the spectroelectrochemical results, chronoamperometric measurements showed largely stable current responses over time (Figure S8), indicating that the IL remains electrochemically robust under the CO_2_RR conditions. The sustained current and the reversible, CO_2_-dependent spectral changes demonstrate that the IL participates dynamically in the electrochemical reaction, while remaining chemically intact. Taken together, the ^1^H NMR and in situ UV–vis data indicate that the IL remains chemically intact during the CO_2_ electrochemical response and does not function as a sacrificial reagent. Instead, the IL responds dynamically to CO_2_ through reversible interactions that alter its electronic and mesoscale properties. Combined with DLS, ATR-IR, and DFT results, these observations show that CO_2_–IL interaction reshapes the local physicochemical environment, facilitating a CO_2_ electrochemical response without permanently transforming the IL.

## Conclusion

In conclusion, this study demonstrates that ILs can function as interfacial media that both support charge transport and facilitate CO_2_ electrochemical response under metal-free conditions. By integrating electrolyte behavior with molecular-level CO_2_ polarization, these ILs facilitate CO_2_ electrochemical response on glassy carbon electrode under aqueous conditions where competitive HER typically dominates. Electrochemical, spectroscopic, and computational analyses show that these ILs interact reversibly with CO_2_, inducing polarization and geometric distortion with implications for lowering the barrier for electron transfer. DFT calculations predict the emergence of a nonzero CO_2_ dipole moment upon interaction with the IL environment, while kinetic isotope effect experiments demonstrate that proton transfer plays a role in the observed electrochemical response. Together, these results suggest a proton-coupled electron transfer framework enabled by IL-mediated modification of the local physicochemical environment. Importantly, spectroscopic analyses before and after redox cycling confirm that the ILs remain structurally intact throughout the process. These observations rule out sacrificial behavior and instead highlight the dynamic, reversible nature of CO_2_–IL interactions, without permanently modifying the IL. Further, systematic variation of IL composition modulates reduction potential and current response, as well as suppresses parasitic HER, underscoring composition as a key design knob for tuning the electrochemical behavior. More broadly, this work refines the understanding of ILs in CO_2_ electrochemical reduction systems. Rather than acting solely as passive electrolytes or cocatalysts, the ILs exhibit a system-specific role in influencing the electrochemical response of CO_2_ in IL-containing electrolytes under the conditions studied. We emphasize that the present work does not address product selectivity or Faradaic efficiency. Instead, it focuses on establishing how ILs influence the local electrochemical environment at the electrode–electrolyte interface through reversible, noncovalent interactions. This interaction-driven approach provides a conceptual basis for simplified, potentially sustainable electrochemical systems and suggests broader opportunities to employ tailored ionic environments to influence the reactivity of other chemically inert small molecules.

## Supplementary Material



## Notes

^1^H and ^13^C NMR spectra of **IL 1** and **IL 2**, cyclic voltammograms of choline and valeric acid controls under CO_2_, ATR-IR spectra of **IL 1** under Ar and CO_2_, UV–vis spectroelectrochemical spectra of Ar-sparged **IL 2**, and chronoamperometric current–time traces of **IL 1** and **IL 2** under CO_2_ conditions (PDF)
