# Supplementary material for: Ionic Liquids as Interfacial Media for Metal-Free Electrochemical CO2 Reduction in Water
Source: ACS Sustain Chem Eng. 2026 Mar 4;14(10):5167–77. doi: 10.1021/acssuschemeng.5c14224 (PMC12997251; doi:10.1021/acssuschemeng.5c14224)
Supplement: Supplementary file 1 [file sc5c14224_si_001.pdf]

Ionic Liquids as Interfacial Media for Metal-Free Electrochemical CO_2 Reduction in Water

Welday Desta Weldu,[1,#] Samuel Abidemi Oluwole,[2,#] Solomon Owiredu,[3] Nicole McGuire,[3] Christian Agatemor[3,*]

[1]Department of Chemistry, Waldorf University, Forest City, IA, 50436, USA.

[2]Department of Chemistry, University of Miami, Coral Gables, 33146, FL, USA.

[3]Department of Chemistry, Bucknell University, Lewisburg, 18737, PA, USA.

[*]Corresponding author: ca024@bucknell.edu

[#]Co-first authors

Number of pages: 6

Number of figures: 8

Number of tables: 0

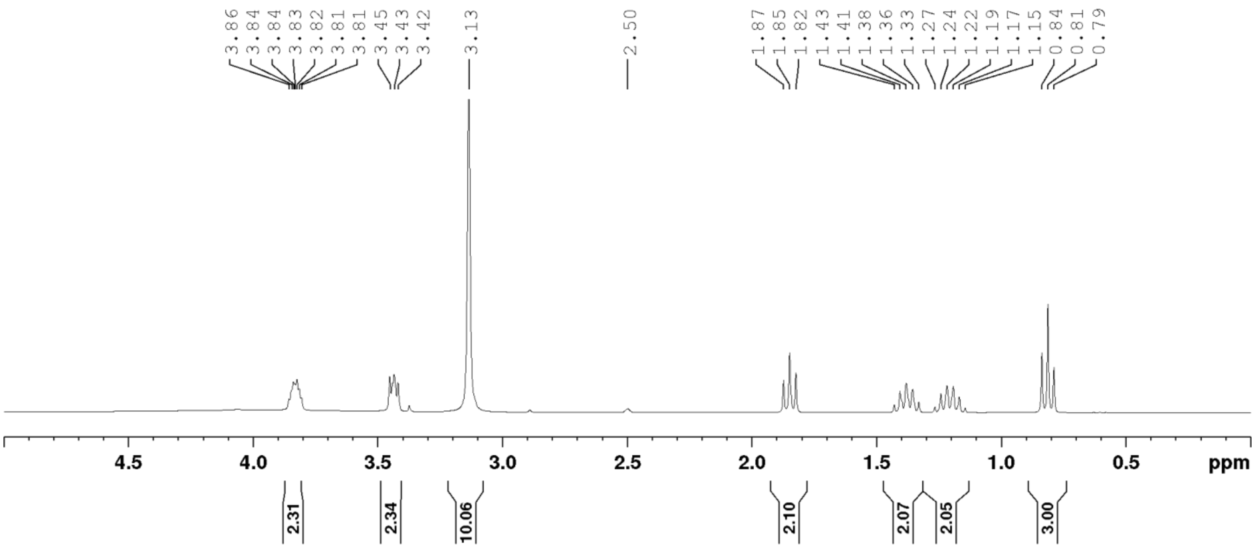

Figure S1. ¹H NMR of **IL 1** (DMSO-d6, 300 MHz).

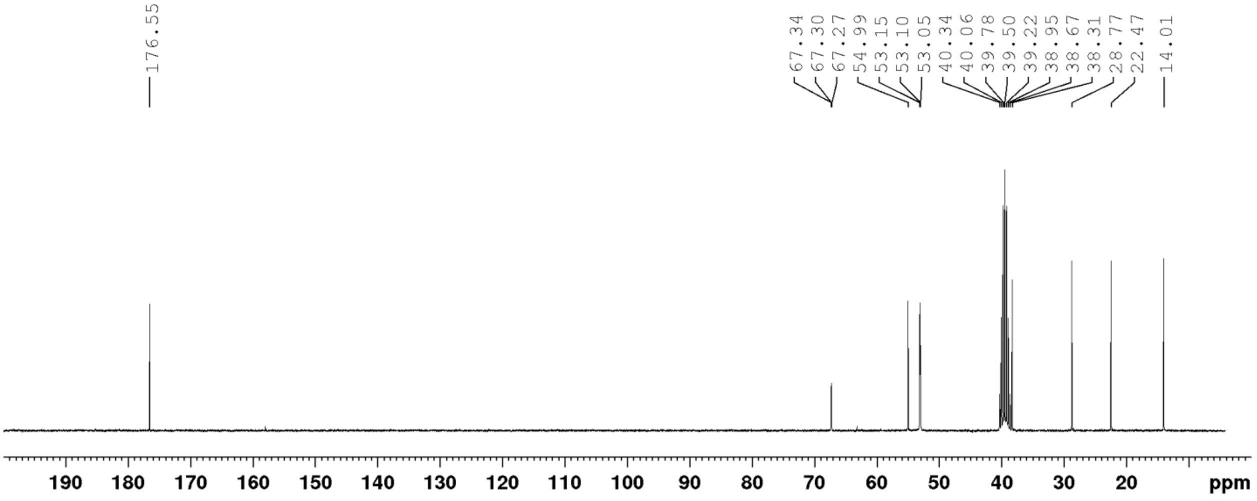

Figure S2. ¹³C NMR of **IL 1** (DMSO-d6, 300 MHz).

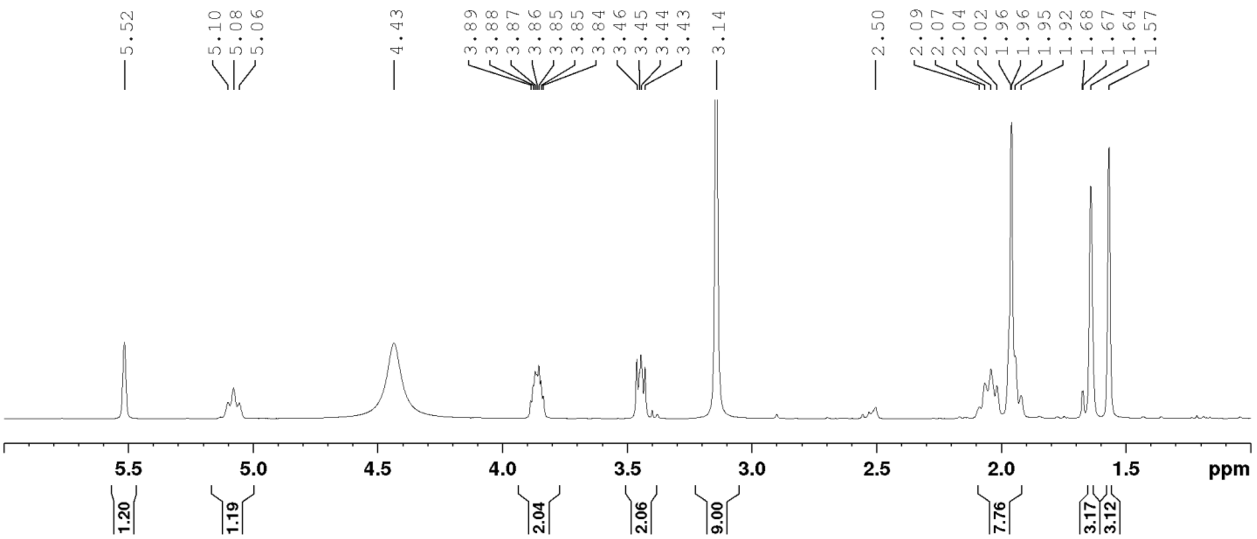

Figure S3. ^{1}H NMR of **IL 2** (DMSO-d$_6$, 300 MHz).

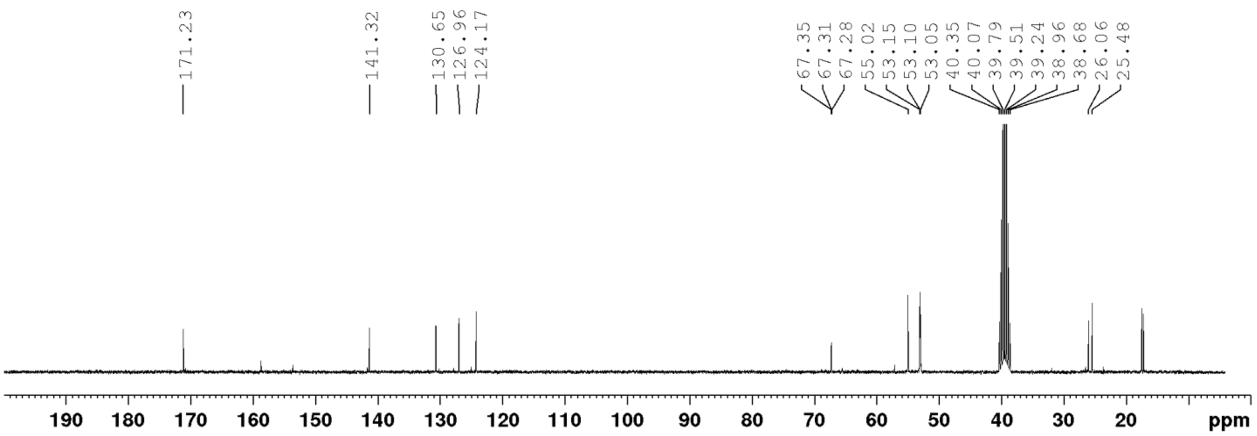

Figure S4. ^{13}C NMR of **IL 2** (DMSO-d$_6$, 300 MHz).

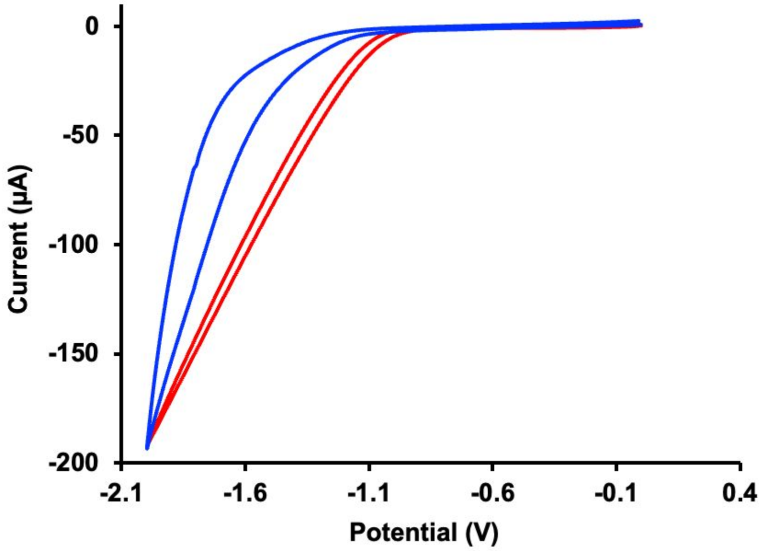

Figure S5. Cyclic voltammograms showing the CO_2RR in CO_2-sparged aqueous solution of choline (blue) and valeric acid (red). The reduction peak was absent, implying choline or valeric acid was unable to facilitate CO_2RR.

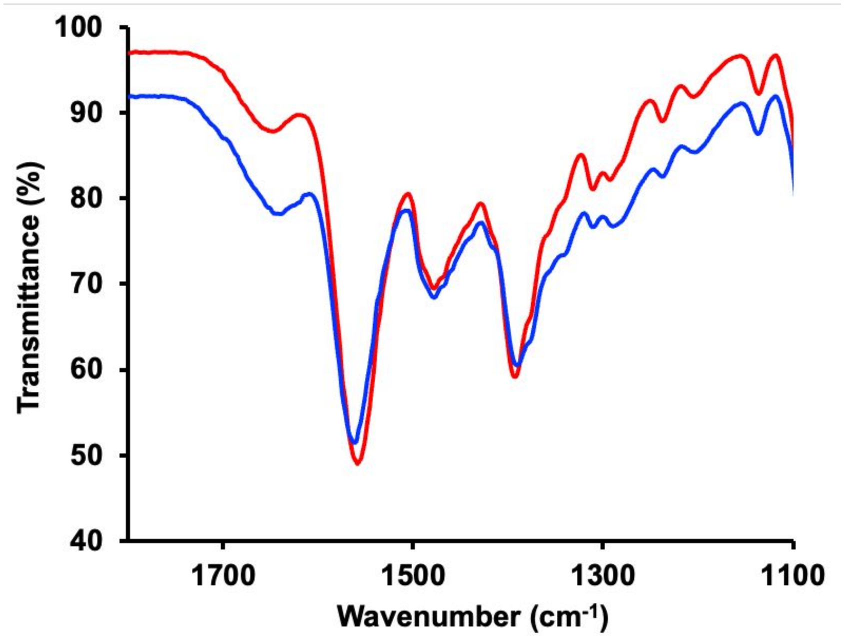

Figure S6. ATR-IR spectra of **IL 1** showing the blue-shifted asymmetric carboxylate C=O stretching mode (~1557 cm^{-1}). Red trace corresponds to argon-sparged **IL 1**, and blue trace corresponds to CO_2-sparged **IL 1**.

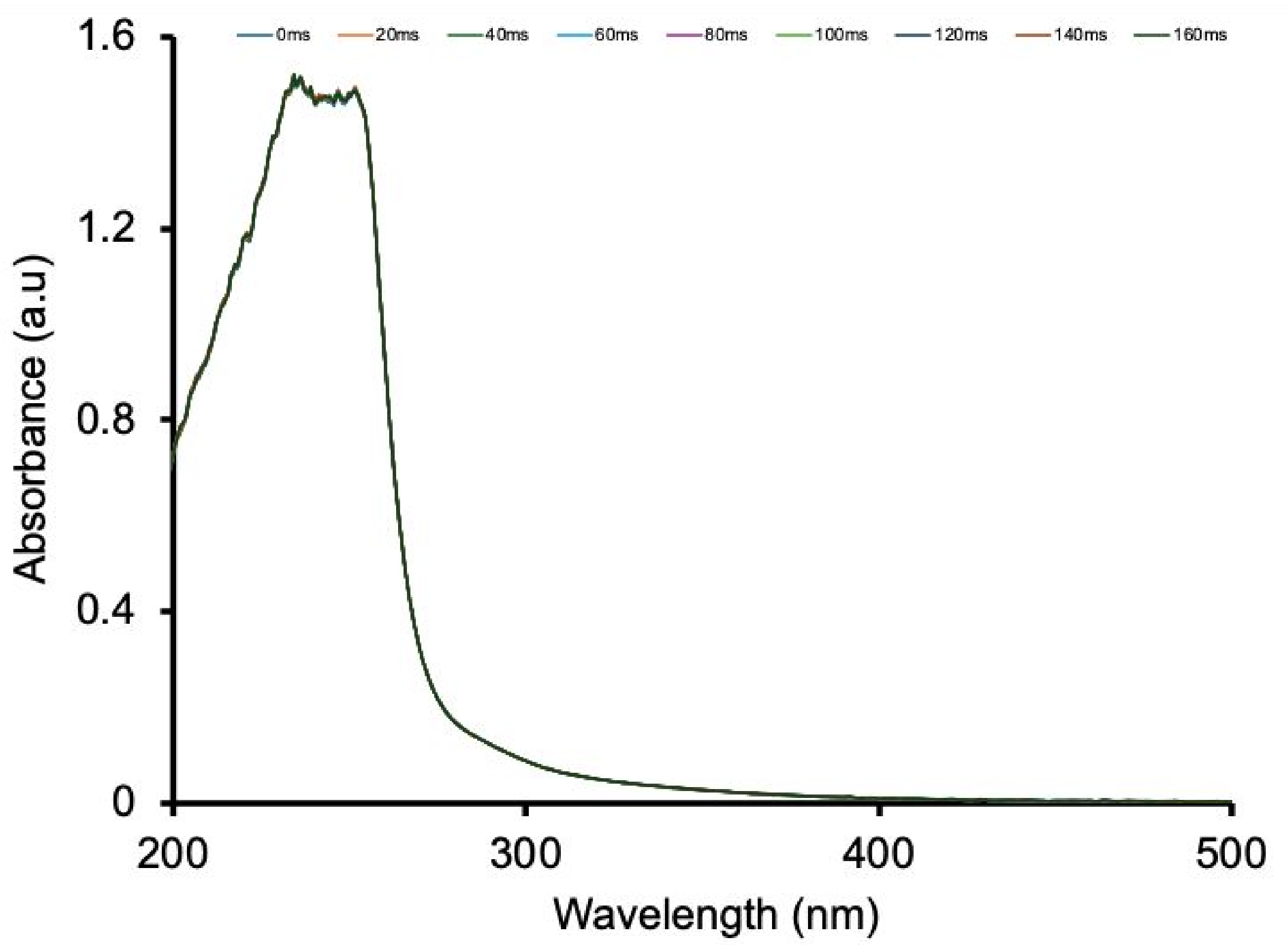

Figure S7. UV–vis absorption spectra recorded during spectroelectrochemical measurements in argon-sparged **IL 2**, illustrating the spectral response as a function of applied potential. The spectra remained unchanged and overlapped throughout the experiment.

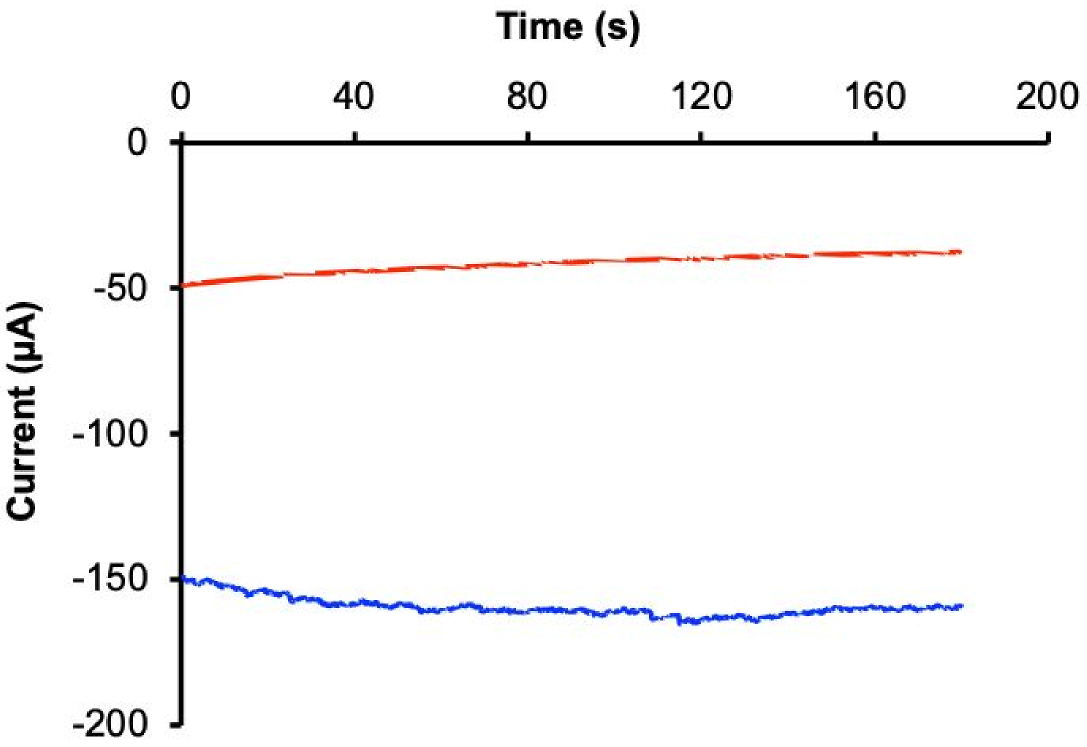

Figure S8. Chronoamperometric responses of ILs under CO_2 conditions. Current–time traces for **IL 2** (blue) and **IL-1** (red), highlight distinct temporal electrochemical behaviors.
